# Flea Burden on Rodents and Its Associated Determinants in Plague‐Endemic Localities of Karatu District, Tanzania: A Cross‐Sectional Study

**DOI:** 10.1002/puh2.201

**Published:** 2024-06-22

**Authors:** Joshua Reuben Jakoniko, Apia Massawe, Elisa Daniel Mwega, Stella Thadeus Kessy

**Affiliations:** ^1^ Department of Veterinary Medicine and Public Health Sokoine University of Agriculture Morogoro Tanzania; ^2^ African Centre of Excellence for Innovative Rodent Pest Management and Biosensor Technology Development Morogoro Tanzania; ^3^ Institute of Pest Management Sokoine University of Agriculture Morogoro Tanzania; ^4^ Department of Veterinary Microbiology Parasitology and Biotechnology Sokoine University of Agriculture Morogoro Tanzania; ^5^ School of Life Science and Bio Engineering Nelson Mandela African Institute of Science and Technology Arusha Tanzania

**Keywords:** ectoparasite, flea, plague‐endemic area, plague foci, rodent, specific flea index

## Abstract

**Background:**

Fleas infest rodents and other small mammals, serving as vectors for zoonotic diseases such as plague. This study investigates the flea burden on rodents and its associated determinants within the plague‐endemic localities of Karatu district, Tanzania.

**Methods:**

A repeated cross‐sectional design was employed to capture rodents with Sherman traps in farmland, peridomestic area, bush land, and forest buffer zones across the wet and dry seasons of 2022 in plague and nonplague foci villages. Captured rodents were anaesthetized and thoroughly brushed to collect fleas, which were then identified using a dichotomous key.

**Results:**

A total of 291 rodents (9 species) were captured, from which 190 fleas (4 species) were collected. The collected fleas were *Dinopsyllus lypusus* (46.32%), *Ctenophthalmus* sp (26.84%), *Xenopsylla brasiliensis* (16.32%), and *Xenopsylla cheopis* (10.53%). Approximately 38.42% of fleas were found on *Mastomys natalensis*, 22.63% on *Lemniscomys striatus*, and 18.42% on *Rattus rattus*. High flea abundance was recorded in farmland and peridomestic areas. The specific flea index (SFI) of *X. cheopis* on *R. rattus* was 1.0 in plague foci and <0.5 in nonplague foci. A generalized linear model revealed significant influences of rodent species, season, habitats, rodent weight, sex, and plague locations on flea abundance. Significant variation was observed between rodent sexes (*p* = 0.009), and a weak positive correlation existed between rodent weight and flea abundance (*R* = 0.17, *p *< 0.05).

**Conclusion:**

Villages in plague foci exhibited higher abundances of fleas in comparison to nonplague foci villages. The SFI results for *X. cheopis* on rats in both types of villages did not surpass critical thresholds. Factors such as dry season, farmlands, and rodent characteristics influenced flea abundance on rodents in the study area.

## Graphical abstract

Fleas infest rodents and other small mammals, serving as vectors for zoonotic diseases such as plague. Villages in plague foci exhibited higher abundances of fleas in comparison to non‐plague foci villages. Factors such as dry season, farmlands, and rodent characteristics influenced flea abundance on rodents in the study area.

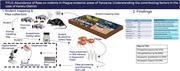

## 1 Introduction

Fleas (*Siphonaptera*) are hematophagous insects and obligatory ectoparasites of vertebrates [1]. They inhabit a variety of habitats, ranging from wet tropical forests to semiarid and desert areas [2, 3]. These insects live an obligate parasitic life with a wide range of potential hosts, primarily infesting small mammals, and, less frequently, birds [4]. They exhibit a holometabolous type of lifecycle, completed within a period of 14–140 days, depending on variations in temperature and humidity in their surroundings [5, 6, 7].

Fleas play a major role in the circulation of zoonotic pathogens among rodent hosts [4]. They have received considerable attention primarily due to their role as vectors of numerous zoonotic diseases, including plague and other emerging pathogens that cause zoonoses, such as bartonelloses, tularemia, and rickettsioses (such as flea‐borne spotted fever, Q fever, and murine typhus), as well as myxomatosis and trypanosomiasis [2]. They can also serve as intermediate hosts for certain helminthes [8]. Nevertheless, the most well‐known flea‐borne zoonotic disease is plague, caused by the bacterium *Yersinia pestis* [9]. This zoonotic bacterium gained notoriety for causing a vast number of human fatalities during the 14th‐century Black Death [10, 11, 12].

Plague remains a persistent threat to the public health in numerous global regions, including African countries [11]. Conducting surveillance studies on flea assemblages within rodent communities in plague‐endemic areas allows for the comprehension of the factors that influence flea abundance and infestation potential on rodents. This comprehensive understanding of these determinants positions proactive strategies to address significant concerns that might pose threats to the public health.

The persistence of plague in endemic areas is characterized by the coexistence of interactions among rodent communities and flea species, with certain species of rodents and fleas demonstrating greater efficiency in the maintenance, amplification, and transmission of *Y. pestis* [4]. *Xenopsylla cheopis* has frequently been identified as a key contributor to the persistence of the plague pathogen in the plague‐endemic areas of Tanzania and other countries [13, 14, 15]. Given its superior efficiency compared to other flea species, *X. cheopis* has become a primary focus for plague risk surveillance, with its specific flea index (SFI) recorded on rats [4]. Other flea species that also pose a threat to public health and facilitate the spread of infection during plague outbreaks include *Xenopsylla brasiliensis* and *Dinopsyllus lypusus*, along with rodent species such as *Lophuromys* spp, *Praomys delectorum*, *Graphiurus murinus*, *Lemniscomys striatus*, *Mastomys natalensis*, and *Rattus rattus* [14, 15, 16].

A study conducted in the plague outbreak focus in the Rift Valley, northern Tanzania [17] has emphasized the critical role of concurrent host–vector interactions in driving the transmission of the plague pathogen. It also highlighted the pivotal role of multiple associations between domestic and peridomestic rodent species infested with fleas, contributing to the persistence and spread of the plague within endemic areas. The dynamics of host‐flea interactions, especially regarding plague maintenance and amplification, are considerably influenced by spatiotemporal changes [18].

Karatu district is an endemic area for plague disease [14, 17]. Previous studies have reported the presence of *Y. pestis* DNA in rodents in the active plague foci of Karatu district, with recorded outbreaks and several cases in 1996 and 1997. The reported prevalence of *Y. pestis* in rodents was 10% in the plague surveillance report of 2006 in this area [17]. This suggests that the area possesses potential attributes for the persistence and transmission of the plague pathogen, justifying the consistent monitoring and surveillance of risk factors for plague outbreaks in the community.

The distribution and community structures of fleas are influenced by numerous biotic and abiotic factors that include host species diversity, sex, age, body size, immune status, host population abundance, habitat diversity, and seasonal variation of temperature and precipitation [19, 20]. Host diversity is a most pertinent factor to consider as it involves variation in flea species richness [21], yet it is not a fixed rule, fleas can infest hosts phylogenetically close, switching between coexisting species within guilds [22].

Given this uncertainty, effective plague management in plague foci often necessitates an inclusive approach that encompasses rodents and their flea parasites. As described here [23], host communities can significantly influence parasitism. The organizational structure of parasite communities is primarily attributed to their hosts, as hosts provide a habitat for parasites, offering a place for living, feeding, and mating [24]. Consequently, a host can be perceived as a biological shelter for parasites. Unlike endoparasites, ectoparasites are influenced not only by host characteristics but also by features of the host environment [25]. Therefore, the habitat of an ectoparasite should encompass not only a specific host but also a specific habitat of that host. In this context, a complex relationship between hosts and habitats becomes a crucial determinant of parasite community structure. Quantifying the variation of flea load indices among host species and analyzing the variation in conjunction with other ecological factors known to shape the host community is a robust approach for understanding the dual nature of host‐parasite interactions [26, 27].

The primary objective of this study was to investigate the flea burden on rodents and its associated determinants within the plague‐endemic localities of Karatu district. The specific objectives aimed to describe: (a) the quantification and comprehensive evaluation of various flea parasitological indices, including the SFI, total flea index (TFI), and flea infestation prevalence on rodents; (b) assessment of the complex interaction between flea species abundance and diverse factors such as rodent species, plague, and nonplague foci villages, seasonal variations (wet and dry), habitat types, and rodent sex; (c) examination of potential flea‐biased parasitism patterns linked to rodent weight and sex (male and female); and (d) analysis of how different host characteristics (including rodent species, sex, and weight), habitat types, seasonal variations (wet and dry season), and specific plague and nonplague foci collectively influence the abundance of fleas in Karatu district.

## 2 Methods

### 2.1 Study Design, Setting, and Sampling

**FIGURE 1:**
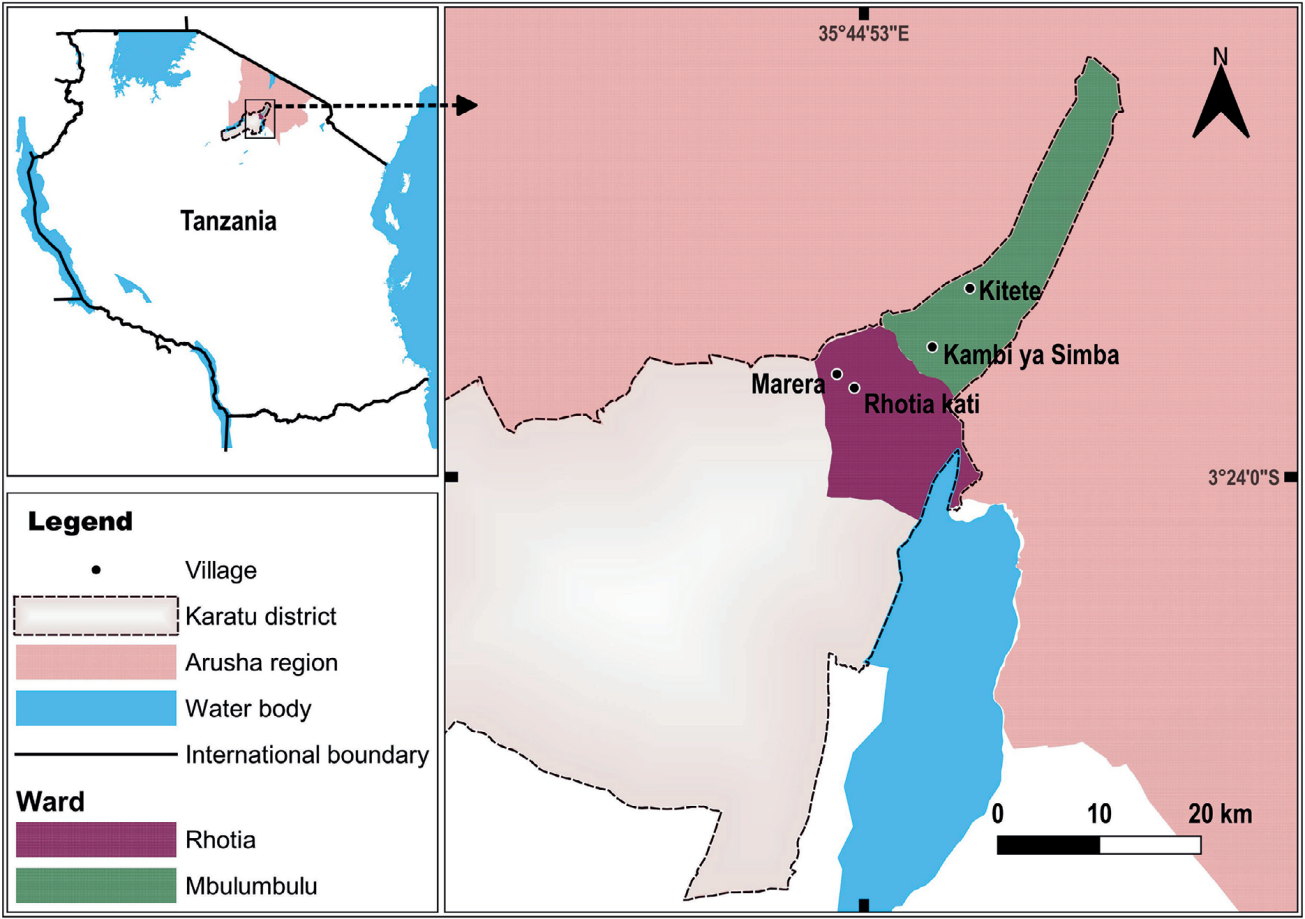
Map of Tanzania (L) highlighting plague foci villages (Rhotia Kati and Kambi ya Simba) (R) and nonplague foci villages (Kitete and Marera) selected for rodent sampling.

This study employed a repeated cross‐sectional design involving the collection of rodent samples across seasons. Rodents were captured using Sherman animal live traps in both plague and nonplague focus villages during wet season, January–February, and dry season, July, 2022. The study took place in the Karatu district, located in the Arusha region, in the northern part of Tanzania. Specifically, it was conducted in four selected villages within the Karatu district: Rhotia Kati, Kambi ya Simba, Kitete, and Marera. Rhotia Kati and Kambi ya Simba are plague focus villages, whereas Marera and Kitete are nonplague focus villages [14] (Figure 1).

### 2.2 Social and Environmental Conditions

Karatu is predominantly inhabited by traditional tribes, who are pastoralists, hunters, and gatherers. Economic activities of residents in the study area involve crop cultivation, livestock keeping, and small retail shops. It is anticipated that in the long term, Karatu Township will become the largest tourist destination after Arusha city on the Northern circuit. The district's climate varies across different areas, with annual rainfall ranging between 900 and 1000 mm. The villages are characterized by short, hot, and long rainy seasons from November to May and a long cold and dry season from June to October. In March to April, the intensity of rainfall can be significant enough to cause severe soil erosion (Source: Leaders at the local study sites, local weather‐forecast:/https://karatudc.go.tz/historia).

These villages were selected depending on the information of whether been involved or not involved in the past human plague outbreak [14]. Rodents were sampled from four habitats, namely, farmland, bush land, peridomestic area, and forest buffer zone that were selected purposefully from each village. This approach aimed to optimize comprehensive representation of the general rodent population within the study area. Village residents assisted in locating areas facing rodent‐related challenges, which greatly contributed to the selection process.

Observations of rodent traces, such as pathways, droppings, burrows, and chewed seeds or rodent trails in the fields, were included as criteria for the selection of the trapping sites and were particularly evident in farmlands and peridomestic areas. Before initiating the trapping, informed consent was sought from the owners of the designated trapping areas. Bush lands, farmlands, and forest buffer zones were all classified as sylvatic areas and were situated at least 300–500 m away from human settlements. Peridomestic areas were identified as the land surrounding human settlements within a range of 100 m.

Rodent sample size was estimated using the sample size formula [28] based on the 10% prevalence of *Y. pestis* in rodents reported in the study area [14]. The estimated rodent sample size was 140, collected twice during wet and dry seasons, resulting in a total sample size of 280 rodents.

### 2.3 Data Collection

#### 2.3.1 Rodent Trapping and Processing

A total of 120 Sherman traps, baited with a mixture of peanut butter and maize flour, were used to capture rodents in both plague and nonplague focus villages across farmland, bush land, peridomestic area, and forest buffer zone. A total of 30 traps were positioned at 10 m intervals along six transect lines, each containing five trapping stations, across all habitats. However, in peridomestic area, 30 Sherman traps were placed at intervals of 2–5 m apart, targeting locations with rodent burrows or other signs of rodent activity around human residences and livestock shelters. The traps were usually set in the field during the evening at 17:00 h and left overnight for three consecutive days, with daily inspections conducted every morning and afternoon to check for captured rodents.

Captured rodents were carefully removed from the traps using an animal handling bag and anaesthetized with diethyl ether applied to a container lined with cotton wool. Then, rodents were brushed to remove fleas, until no more fleas fell into the plastic basin [15]. Animal handling bag and the container were checked for the presence of fleas which were all collected with a fine forceps. Collected fleas were counted, recorded, and preserved in a well‐labeled Eppendorf tubes containing 70% ethanol for morphological identification. Rodents were taxonomically identified to the genus and species levels following an established nomenclature for rodent identification [29]. Finally, rodents were euthanized with diethyl ether in a container for the collection of tissue biopsy for further analyses (unpublished data).

#### 2.3.2 Fleas Processing and Identification

Fleas were processed in the laboratory following the standard procedure outlined in Refs. [30, 31]. This procedure involved subjecting fleas in a series of reagents to enhance their features, making them distinct enough for identification using a dichotomous key. Subsequently, the processed fleas were mounted on microscope glass slides [32] and observed under a digital stereo microscope OPTA‐TECH for identification into genus and species levels, following the conventional dichotomous key method [33, 34]. Specimens of each identified flea species were preserved at the College of Veterinary Medicine and Biomedical Sciences—SUA.

#### 2.3.3 Data Analysis

Flea data were recorded and organized into a readable dataset format. This dataset was then imported in R programming language software (R Core Team version 4.2.2 of 2022) for all statistical analyses, with a significance level (*α*) of 0.05. Prior to analysis, data were tested for normality and were found to be not normally distributed (Shapiro–Wilk test *p* < 0.05), and nonparametric tests were then employed for inferential statistics. The assessment of rodent flea loads on examined rodents was conducted through the evaluation of total flea indices and specific flea indices, following the methodology outlined in Ref. [35]. The percentage prevalence of rodents infested with fleas (i.e., the proportion of examined rodents with fleas) was computed along with a 95% confidence interval (CI) of the proportion.

Chi‐square test was performed to examine the relationship of abundance of species of fleas with rodent species, plague and nonplague foci villages, season, habitat type, and rodent sex. The nature of association was further analyzed to find the contribution of level of factors to the chi‐square result using absolute standardized residuals. The outcomes of this analysis were then visually depicted using a balloon chart [36].

The phenomenon of flea‐biased parasitism in relation to rodent sex and weight was examined using the Wilcoxon rank sum test and the Spearman rank correlation test (rho), respectively. The Wilcoxon rank sum test was employed to compare flea abundance between rodent sexes, whereas Spearman rank correlation test (rho) was employed to assess the strength and direction of association between flea abundance and rodent weight; building upon this, further analysis was conducted using generalized linear model (GLM) to determine the influence of these factors on flea abundance. The GLM was selected as the analytical approach to establish the model variables that elucidate the influence of predictors on flea abundance. The model was selected based on AIC, with the model having the possible lowest AIC being selected using the step‐AIC function in the MASS package of the R programming language [37]. Predictor variables for this analysis involved both numerical and categorical variables, which include host characteristics (rodent species, sex, and weight), habitats, season, and plague localities (plague and nonplague foci villages).

#### 2.3.4 Ethical Approval

This study was approved by the Institutional Review Board of Sokoine University of Agriculture, Tanzania (Ref. No. SUA/DPRTC/R/186/15).

## 3 Results

### 3.1 Flea Collected from Rodents

Four species of fleas (*n* = 190) were collected from six species of rodents (*n* = 73) out of a total of nine rodent species (*n* = 291). The TFI fluctuated across various temporal and spatial conditions. Specifically, we observed a TFI of 0.53 (SD = 1.54) during the wet season, which increased to 0.78 (SD = 1.66) in the dry season. Upon closer examination of focal distribution, plague foci villages exhibited a TFI of 0.71 (SD = 1.81), whereas nonplague foci regions displayed a comparatively lower index of 0.55 (SD = 1.13). The flea species collected in this study were *D. lypusus* (46.32%), *Ctenophthalmus* spp (26.84%), *X. brasiliensis* (16.32%), and *X. cheopis* (10.53%). These collected fleas were evaluated to estimate both the TFI and the prevalence of flea infestation among rodent species (see Table 1).

**TABLE 1:** Total flea index (TFI) and percentage prevalence of infestation in rodents sampled from Karatu district, Arusha region, Tanzania, 2022.

Rodent species	Rodents examined (*n* %)	Rodents infested with fleas (prevalence)	Fleas collected (*n* %)	TFI
*Arvicanthis niloticus*	70 (24.05)	12 (0.17)	27 (14.21)	0.39
*Grammomys* spp	12 (4.12)	0	0	0
*Graphiurus murinus*	2 (0.69)	1 (0.5)	3 (1.58)	1.5
*Lemniscomys striatus*	43 (14.78)	13 (0.3)	43 (22.63)	1
*Lophuromys flavopunctatus*	13 (4.47)	4 (0.31)	9 (4.74)	0.69
*Mastomys natalensis*	122 (41.92)	31 (0.25)	73 (38.42)	0.6
*Otomys* spp	3 (1.03)	0	0	0
*Praomys delectorum*	1 (0.34)	0	0	0
*Rattus rattus*	25 (8.60)	12 (0.48)	35 (18.42)	1.4
**Total**	**291**	**73 (0.25)**	**190**	**0.65**

It is worth noting that the sampling effort in this study did not yield any fleas from three distinct rodent species: *Grammomys* spp*, P. delectorum*, and *Otomys* spp. This absence could be attributed to the very low abundance of these captured rodents, thereby reducing the likelihood of capturing individuals infested with fleas.

### 3.2 Variation of Flea Infestation Prevalence on Rodent Community

It was found that the estimated prevalence of rodents infested with fleas was 25% (CI = 20–30), which was significantly lower than the 50% expected infestation prevalence on rodents in plague‐endemic area (*p* < 0.001). Variations in the prevalence of rodents infested with fleas were observed across different habitats, seasons, and plague and nonplague foci villages. Remarkably, farmland and peridomestic areas exhibited high flea prevalence, which was 30% (CI = 21–40) and 26% (CI = 18–35), respectively, compared to forest buffer zone and bush land, which was 24% (CI = 14–38) and 17% (CI = 9–29), respectively. Moreover, plague foci villages demonstrated a high flea prevalence of 26% (CI = 20–33), whereas nonplague foci villages exhibited a prevalence of 23% (CI = 16–32). The dry season exhibited a high flea prevalence of 30% (CI = 23–38), contrasting with the wet season prevalence of 20% (CI = 15–27).

### 3.3 Specific Flea Indices (SFI) on Rodents in Different Habitats

Fleas collected from all rodent species were integral to the determination of specific animal flea indices across diverse habitats (see Table 2). *D. lypusus* exhibited infestations in five rodent species, encompassing 42.47% of all rodents infested with fleas. *Ctenophthalmus* spp infested four rodent species equal to 32.88% of all rodents infested with fleas, *X. brasiliensis* was collected from three rodent species equal to 12.33%, whereas *X. cheopis* was found to infest two rodent species equal to 12.33% of all rodents infested with fleas.

**TABLE 2:** Specific flea indices (SFI) across different habitats in rodents sampled from Karatu district, Arusha region, Tanzania, 2022.

				Fleas collected and (SFI)			
Habitats	Rodent species	Rodents examined	Rodents infested (prevalence)	*Xenopsylla brasiliensis*	*Xenopsylla cheopis*	*Dinopsyllus lypusus*	*Ctenophthalmus* spp
**Bush land**	*Arvicanthis niloticus*	27	4 (0.15)	0	0	6 (0.22)	3 (0.11)
	*Grammomys* spp	5	0	0	0	0	0
	*Lemniscomys striatus*	7	0	0	0	0	0
	*Mastomys natalensis*	12	5 (0.42)	0	0	4 (0.33)	1 (0.08)
	*Otomys* spp	2	0	0	0	0	0
**Farm land**	*A. niloticus*	10	2 (0.2)	0	0	4 (0.4)	0
	*Grammomys* spp	1	0	0	0	0	0
	*Lemniscomys striatus*	29	10 (0.34)	4 (0.14)	0	19 (0.66)	12 (0.41)
	*Lophuromys flavopunctatus*	2	1 (0.5)	0	0	2 (1)	0
	*Mastomys natalensis*	45	13 (0.29)	3 (0.07)	3 (0.07)	16 (0.36)	15 (0.33)
**Forest buffer zone**	*Grammomys* spp	5	0	0	0	0	0
	*Graphiurus murinus*	2	1 (0.5)	0	0	3 (1.5)	0
	*L. flavopunctatus*	11	3 (0.27)	0	0	6 (0.55)	1 (0.09)
	*M. natalensis*	27	7 (0.26)	6 (0.22)	0	8 (0.3)	3 (0.11)
	*Praomys delectorum*	1	0	0	0	0	0
**Peridomestic**	*A. niloticus*	33	6 0.18)	0	0	5 (0.15)	9 (0.27)
	*Grammomys* spp	1	0	0	0	0	0
	*L. striatus*	7	3 (0.43)	0	0	7 (1)	1 (0.14)
	*M. natalensis*	38	6 (0.16)	0	0	8 (0.21)	6 (0.16)
	*Otomys* spp	1	0	0	0	0	0
	*Rattus rattus*	25	12 (0.48)	18 (0.72)	17 (0.68)	0	0
	**Total**	**291**	**73 (0.25)**	**31**	**20**	**88**	**51**

### 3.4 Variation of Specific Flea Indices (SFI) on Rodents in Plague and Non‐plague Foci

**FIGURE 2:**
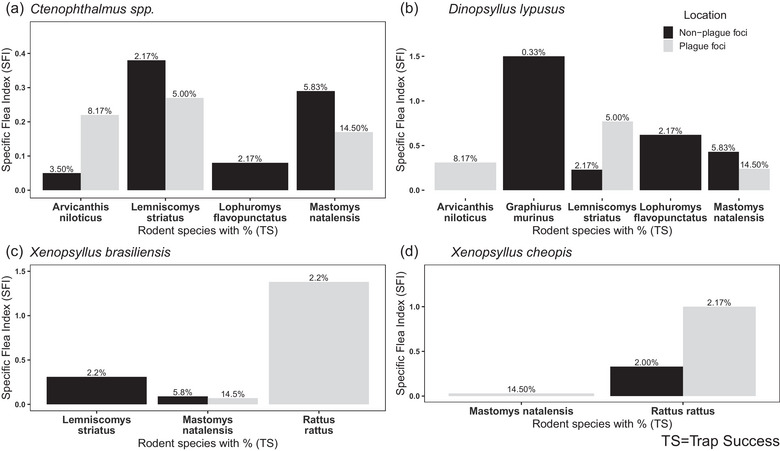
Comparison of specific animal flea index (SFI) on rodent species collected in plague and nonplague focus villages in plague‐endemic area of Karatu district northern Tanzania, 2022. The percentage represents trap success of each individual rodent.

In nonplague foci villages, rodents were primarily infested with *Ctenophthalmus* spp and *D. lypusus*, as depicted in Figure 2a,b. *Ctenophthalmus* spp exhibited high infestations in two rodent species, namely, *L. striatus* SFI 0.39 and *M. natalensis* SFI 0.3, whereas *D. lypusus* showed preference in *G. murinus* compared to other captured rodent species SFI 1.5. Conversely, in plague foci villages, the majority of captured rodents were infested with *X. brasiliensis* and *X. cheopis*, as illustrated in Figure 2c,d. Among these, *X. brasiliensis* and *X. cheopis* were predominantly associated with *R. rattus* SFI 1.48 and SFI 1.0, respectively, compared to other rodent species.

### 3.5 Association of Abundance of Flea Species with Other Factors

**FIGURE 3:**
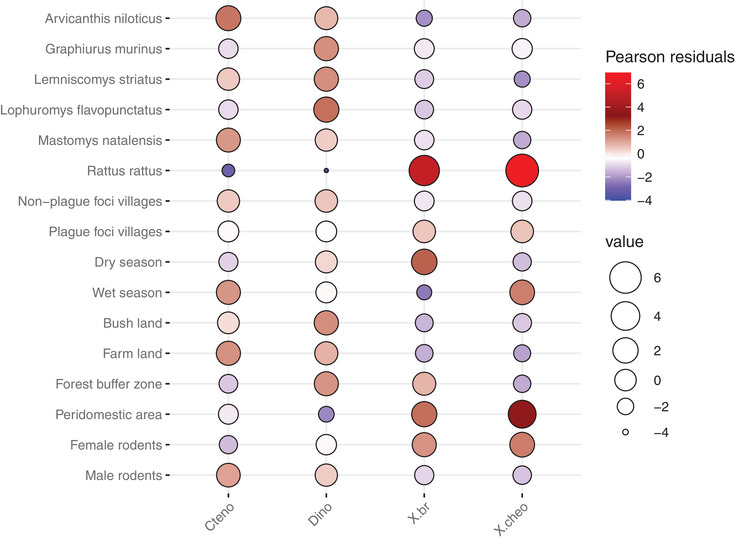
The nature of the association of the abundance of flea species with each level of each factor using the standardized chi‐square residuals (Pearson residual) to determine the major contributors of the significant result per factor. *Cteno*, *Ctenophthalmus* spp; *Dino*, *Dinopsyllus lypusus*; *X. br*, *Xenopsylla brasiliensis*; *X. cheo*, *Xenopsylla cheopis*.

The abundance of flea species was found to be statistically significantly associated with the type of habitat (*p* < 0.001), season (*p* < 0.001), rodent species (*p* < 0.001), and rodent sex *p* = 0.014). However, it was not statistically significantly associated with plague locations (plague and nonplague foci) (*p* = 0.49). A balloon chart was plotted to depict these relationships using Pearson residuals (standardized residuals) for all levels of each factor. When the absolute value of the residual exceeds 2.00, it indicates that the level of the particular factor significantly contributes to the chi‐square test statistic, and thus, it is significantly associated with the abundance of corresponding flea species. High pearson residual values were represented by large and bright‐red balloons in a gradient of size and color, with medium‐sized balloons in dark‐red and small balloons in pale‐red. This gradient illustrated the strength of the positive association between specific levels of the factor and the abundance of particular flea species. The sequence of large, bright‐red balloons followed by dark‐red balloons indicated a strong positive relationship between particular level of the factor and the abundance of particular flea species. This relationship weakens as the size of the balloons decreases. Meanwhile, the gradient of smaller balloons ranges in color from white (indicating no relationship) to pale and bright blue (indicating a negative relationship) (see Figure 3).

### 3.6 Flea Biased Parasitism on Rodent's Sex and Weight

**FIGURE 4:**
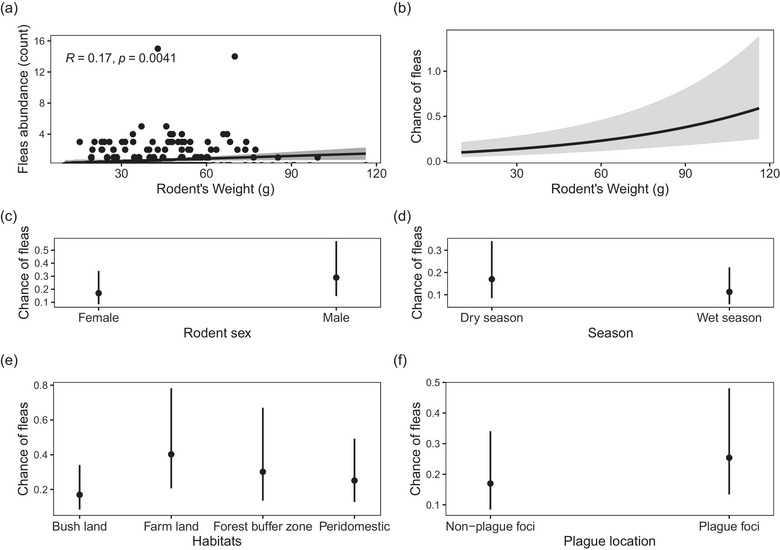
(a) Spearman's rank correlation test of rodent weight and flea abundance. (b) Generalized linear model (GLM) prediction curve illustrating the influence of rodent weight on flea abundance. (c–f) GLM categorical predictor variables and their influence on flea abundance.

A notable difference in flea abundance was evident between male and female rodents (Wilcoxon rank sum test W = 9158.5, *p* = 0.009), with a small magnitude of effect size (*r* = 0.153). The median weight for each sex was 40.1 g (IQR = 25.3 g) for males and 39.4 g (IQR = 23 g) for females; however, the difference was not statistically significant (W = 10140, *p* = 0.54). Furthermore, a significant correlation between flea abundance and rodent weight was observed within the captured rodent populations in the study area. This correlation was unveiled by Spearman's rank correlation coefficients (*R* = 0.17, *S* = 34176, *p* < 0.05), highlighting a subtle yet affirmative connection (Figure 4a).

### 3.7 Factors Influencing Flea Abundance

The best fitting model derived from the GLM was statistically significant in elucidating the relationship between predictor variables and flea abundance (*χ* ^2^(15) = 106.63, *p* < 0.001), as shown by the model coefficients in (Table 3). The graphical chart of predictors is displayed in Figure 4 (panels b–f), illustrating the probability of fleas in various factors.

**TABLE 3:** Summary of the best fitting coefficients of generalized linear model (GLM) (Poisson family) describing the influence of predictor variables (parameters) on abundance of fleas.

Parameters	Estimate	Std. error	*Z* value	*p*‐Value
Intercept	−2.48	0.41	−6.05	<0.001
Rodent species				
*Grammomys* spp	−14.96	573.36	−0.03	0.98
*Graphiurus murinus*	1.40	0.72	1.96	0.05
*Lemniscomys striatus*	0.56	0.29	1.91	0.06
*Lophuromys flavopunctatus*	0.47	0.50	0.95	0.34
*Mastomys natalensis*	0.24	0.25	0.93	0.35
*Otomys* spp	−14.91	1206.53	−0.01	0.99
*Praomys delectorum*	−15.46	2103.36	−0.01	0.99
*Rattus rattus*	1.34	0.29	4.66	<0.001
Season				
Wet season	−0.41	0.19	−2.15	0.03
Habitats				
Farm land	0.86	0.31	2.79	0.005
Forest habitat	0.58	0.38	1.50	0.13
Peridomestic	0.39	0.32	1.21	0.23
Rodent weight	0.017	0.004	4.20	<0.001
Rodent sex				
Male	0.53	0.15	3.46	<0.001
Plague location				
Plague foci villages	0.40	0.18	2.20	0.03

## 4 Discussion

### 4.1 Flea Species Collected from Rodents

Four species of fleas *X. brasiliensis, X. cheopis, D. lypusus*, and *Ctenophthalmus* spp that were collected from rodent community in both plague and nonplague foci villages are all considered potential or likely to be potential for harboring and transmitting plague infection from rodent to rodent or rodent to human being during the past human plague outbreak in Karatu plague‐endemic area [14, 17]. This consideration extends beyond the Karatu plague‐endemic area to other districts in the country, which also harbor potential areas for plague persistence [15, 17, 38]. The most efficient flea vector for the transmission of plague infection is *X. cheopis*, which plays a major role in the circulation of plague bacilli during both enzootic and epizootic plague [16].

The high vectoring potential of *X. cheopis* is attributed to its distinctive proventricular spines, which offer an attachment site for *Y. pestis* colonization. This leads to the formation of a proventricular blockage after the flea feeds on an infected host. As a result, *X. cheopis* increases its daily biting rate to acquire blood meals, while regurgitating multiple times at the bite site in an attempt to clear its blocked proventriculus. This behavior facilitates the higher transmission of infection compared to other flea species [39, 40].

*D. lyppusus, X. brasiliensis*, and *Ctenophthalmus* spp. are considered enzootic plague vectors, facilitating the rapid transmission of the disease pathogen during plague outbreaks [16, 41, 42]. *D. lyppusus* and *X. brasiliensis* were identified as competent vectors for transmitting the plague disease during the 2007 plague outbreak in Mbulu and the 1996/97 outbreak in the Karatu plague‐endemic area [14, 15]. *D. lyppusus* has been reported in East Africa as a plague vector and an important enzootic flea, circulating *Y. pestis* among rodents. These reports date back to the 20th century following the introduction of the disease, as documented by Refs. [43, 44, 45]. This suggests that the collected flea species, particularly *X. brasiliensis, X. cheopis*, and *D. lyppusus*, play crucial roles in the circulation of the plague pathogen among rodents and other potential small mammals, thereby facilitating the persistence and maintenance of plague bacilli within the plague‐endemic foci of the Karatu district.

### 4.2 Specific Flea Index

Oriental rodent fleas (*X. cheopis*) have been a subject of significant concern within the realm of public health due to their efficiency in amplifying and transmitting *Y. pestis* from rodent to rodent and from rodents to humans [16, 46]. In light of this concern, an assessment of SFI was conducted, recognizing its potential significance as an indicator of risk of plague outbreak among rodents and the surrounding society [47, 48, 49]. Notably, an SFI >1 for *X. cheopis* on rats has been identified as an indicator of a potentially dangerous situation, signifying an increased risk of human plague transmission during an outbreak of the disease [47]. Moreover, the likelihood of a human plague outbreak is higher if the SFI is >5 [50].

Based on the findings derived from this study, it has been observed that the SFI of *X. cheopis* exhibited parity with unity in plague focus villages, higher compared to nonplague focus villages. Although the observed indices may not warrant immediate interventions for flea control within these villages, they, nevertheless, offer the fundamental insights into the dynamics of the infection, highlighting the need for increased attention in plague focus villages to prevent escalation of the situation beyond acceptable risk levels. The increased SFI in plague foci villages can plausibly be attributed to the augmented abundance of hosts of the oriental rodent fleas, notably *R. rattus*, and to a lesser extent, *M. natalensis* in contrast to nonplague foci‐villages, as reported in this study.

### 4.3 Associated Factors Influencing Flea Abundance on Rodents

Essentially, this study revealed that the abundance of fleas within the local rodent community was affected by seasonal fluctuations (wet and dry), plague and nonplague foci villages, variations in habitat types, and host‐specific characteristics. Among these influencing factors, the fluctuations in seasonal dynamics have emerged as a predominant driver of flea abundance. This influence is rooted in the seasonal patterns that govern the distribution and abundance of the fleas’ biological habitats, primarily the rodent populations [51]. The dynamic interplay of temperature and humidity across the changing seasons governs the developmental progression and survival of juvenile fleas [42]. A corpus of studies consistently accentuates the impact of seasonal changes on the abundance of both rodent and flea populations, thereby resonating in the sphere of maintenance and transmission of the plague pathogen in plague‐endemic areas [52, 53, 54, 55].

### 4.4 Season

Variations in wet and dry seasons have been reported to affect the growth, reproduction, and survival of the immature stages of fleas [56, 57]. It was observed that the lower abundance of fleas during wet season was mostly attributed to the increase in soil moisture, which has been speculated to decrease the survival of fleas in rodent burrows [58]. As noted, wet conditions foster the growth of harmful fungi when humidity is over 95%, especially when combined with organic matter, thereby decreasing the viability of larval survival and flea egg fecundity [16]. Conversely, the increase in flea abundance during dry season was likely due to an increase in the abundance of their rodent hosts, coupled with supportive weather conditions characterized by warmth and low humidity during early dry season [42, 58].

### 4.5 Plague and Non‐plague Foci

The abundance of fleas in plague focus villages exhibited a substantial increase compared to nonplague focus villages. This can be ascribed to the considerably large proportion of rodent populations within plague foci villages, providing a multitude of potential biological niches and a consistent reservoir of blood meals for fleas. The increase in rodent abundance can directly influence the increase in the abundance of fleas, thereby contributing to an augmentation in their distribution [54, 59].

### 4.6 Habitats

The variation of habitats was observed as another factor influencing the abundance of fleas in the study area. Different habitats have distinct characteristics that are crucial in determining the distribution and abundance of both rodent hosts and fleas. The change in habitats affects the composition of rodent species and their fleas, making it an important factor in the ecological surveillance of flea abundance [60, 61]. This study has found a significant increase in the abundance of fleas in farmland compared to other habitats. The farmland habitat was observed to encourage rodent colonization, as it promotes the availability of food left after harvest during the dry season. Observations revealed that rodents prefer to reside in areas with abundant food, where they can create burrows and nests to protect themselves and their young from predators. Habitats distinguished by copious food availability, minimal disruption, and limited control measures facilitate the proliferation of rodent populations alongside their concomitant flea cohorts.

### 4.7 Rodent Species

Furthermore, the study observed the differences in flea species infestation on rodents contributed by different rodent species. *R. rattus* showed a significantly higher increase in influencing the abundance of *X. cheopis*, especially in human habitats (peridomestic areas), compared to other rodents. The characteristics of individual rodent hosts have been documented to play a role in the mechanism of flea acquisition [62]. The behavior and inclination of *R. rattus* to inhabit human habitats facilitate frequent interactions and the exchange of fleas among them, given the limited space within this habitat. The movement of *R. rattus* between houses predisposes these individual rodents to encounter numerous fleas, which are subsequently dispersed among other individual rodents within the area. The tactics employed by fleas and other ectoparasites to inhabit microhabitats of host species, such as rodent burrows, or to await the presence of a suitable host, facilitate easy infestation, especially when a new host has visited the burrow [63].

### 4.8 Flea Biased Parasitism on Rodent's Sex and Weight

Flea sex‐biased parasitism was more evident on male rodents compared to females. Male rodents exhibited a significant increase in flea infestation load. This is attributed to the behavior of male rodents, having larger home ranges with broader dispersal areas, prompted by their engagement in visiting multiple female burrows/nests while in pursuit of mates and foraging for sustenance [64]. The phenomenon is explained by the fact that many arthropods, especially sticking ectoparasites, tend to exhibit male‐biased parasitism because most of them wait for the host to visit their area rather than actively seeking out the host [65]. It is important to note that the phenomenon of sex‐biased parasitism cannot be universally generalized. Other studies have demonstrated cases in which both male and female rodent hosts were equally infested with fleas [66].

Similarly, the weight of rodents in the general rodent community studied appears to have a noteworthy correlation with the increasing flea infestation among rodents. Amidst natural phenomena, the increase in rodent weight aligns with the increase in their body size and age. These parameters exert influence over the physiological and behavioral qualities of individual rodents within specific ecological habitats, potentially fostering increased exploration and enabling enhanced survival strategies for sustenance, mates, or improved shelter within their natural environment, thereby increasing the chance of encountering many parasites including fleas. It has been documented that larger rodents are more susceptible to ectoparasites compared to smaller ones, whereas adult rodents tend to tolerate a higher flea burden; juveniles are more inclined to engage in frequent antiparasitic grooming [67].

### 4.9 Recommendations

It is recommended that local communities continue implementing rodent and flea control measures in their vicinity to limit interactions between these main plague agents and the human population, particularly in areas of high human activity such as farmland and peridomestic areas.

Moreover, this study underscores the necessity of maintaining regular surveillance on rodent and flea populations in potential plague‐endemic areas. This approach is crucial for diligently overseeing and executing monitoring strategies intended for plague prevention within the community, while also allowing for adaptations and enhancements as required.

## 5 Conclusion

Villages in plague foci exhibited higher abundances of fleas, thereby increasing the potential for plague maintenance and circulation among rodent populations, in comparison to nonplague foci villages. The SFI results for *X. cheopis* on rats in both types of villages did not surpass critical thresholds. Factors such as dry season, farmlands, and rodent characteristics influenced flea abundance on rodents in the study area.

## Author Contributions

**Joshua Reuben Jakoniko**: conceptualization; data curation; formal analysis; visualization; writing–original draft; methodology; investigation; writing–review and editing; software; project administration; supervision; validation. **Apia Massawe**: supervision; project administration; resources; funding acquisition; validation; investigation; visualization; data curation; formal analysis. **Elisa Daniel Mwega**: data curation; formal analysis; visualization; investigation; supervision; project administration; validation; resources. **Stella Thadeus Kessy**: conceptualization; data curation; formal analysis; visualization; methodology; writing–review and editing; software; validation.

## Conflicts of Interest

No one declared conflicts of interest in this study.

## Data Availability

The authors commit to depositing the data linked to this study in a Mendeley data repository and ensuring its public availability. Data reference link: Jakoniko, J., Massawe, A., Mwega, E., Kessy, S., (2023), “Flea Burden on Rodents and Its Associated Determinants in Plague‐Endemic Localities of Karatu District, Tanzania: A Cross‐Sectional Study”. Mendeley, v1, https://doi.org/10.17632/mydn68pyzz.
